# Exploring the respiratory efficacy of combined chronic glucocorticoid and antioxidant interventions in the *mdx* mouse: The PREDNAC trial

**DOI:** 10.1113/EP092491

**Published:** 2025-05-26

**Authors:** Michael N. Maxwell, Ben T. Murphy, Fiona B. McDonald, Ken D. O'Halloran

**Affiliations:** ^1^ Department of Physiology University College Cork Cork Ireland

**Keywords:** diaphragm, glucocorticoid, inspiratory pressure, *mdx*, muscular dystrophy, respiratory EMG

## Abstract

Duchenne muscular dystrophy (DMD) is characterized by respiratory muscle injury and weakness, ultimately leading to respiratory failure. Impaired respiratory muscle performance, fibrosis and inflammation in early disease are evident in the dystrophin‐deficient *mdx* mouse model of DMD. Prednisone or similar treatment is the current standard of care for DMD and exerts its benefits via an anti‐inflammatory action, but chronic treatment is associated with side‐effects. A recent study demonstrated improved function in *mdx* limb muscle with weekly glucocorticoid treatment compared with daily treatment. Herein, we investigated the effect of weekly α‐methylprednisolone (PRED) treatment alone and the effect of PRED in combination with daily intake of the antioxidant *N*‐acetyl cysteine, NAC (PREDNAC) on respiratory performance. One‐month‐old male *mdx* mice received PRED (0.8 mg/kg methylprednisolone i.p. weekly) or PREDNAC (0.8 mg/kg methylprednisolone i.p. weekly and 1% NAC in drinking water daily) for 3 months. At 4 months of age, conscious breathing was measured in vivo by whole‐body plethysmography. Under urethane general anaesthesia, respiratory EMG and inspiratory pressure were measured at baseline and during maximal activity. The intrinsic force‐generating capacity of the diaphragm was determined *ex vivo*. Neither PRED nor PREDNAC influenced breathing or diaphragm force‐generating capacity in *mdx* mice. There was a significant increase in diaphragm and parasternal EMG activity, but inspiratory pressure was unchanged with treatment. We conclude that neither PRED nor PREDNAC has a major beneficial effect on respiratory system performance in the *mdx* mouse model of DMD. Weekly administration of glucocorticoids is inadequate to protect respiratory performance in *mdx* mice, which might reflect the higher duty cycle of respiratory muscles compared with limb muscles.

## Abstract (highlights)

- **What is the central question of this study?**
  Does combined chronic glucocorticoid and antioxidant treatment improve respiratory system performance in the *mdx* mouse model of Duchenne muscular dystrophy?
- **What is the main finding and its importance?**
  We determined that combined glucocorticoid and antioxidant treatment for 3 months exerted an anti‐inflammatory effect on the *mdx* diaphragm and modestly increased diaphragm peak EMG activity. However, combined therapy did not improve a range of respiratory‐related parameters in *mdx* mice, suggesting that at the doses used in this study there is limited potential for weekly glucocorticoid treatment with or without antioxidant supplementation as an intervention to support respiratory performance in Duchenne muscular dystrophy.

## 1 INTRODUCTION

Duchenne muscular dystrophy (DMD) is a life‐limiting X‐linked neuromuscular disease arising from mutations in the *DMD* gene, leading to the absence of the structural protein dystrophin (Aartsma‐Rus et al., 2006; Hoffman et al., 1987). Dystrophin is integral to the dystrophin glycoprotein complex, a structure that links F‐actin in the myocellular cytoskeleton to the extracellular matrix via the sarcolemma, thus offering structural support during repetitive contraction and protecting muscle fibres (Gao & McNally, 2015). In DMD, there is a complete absence of dystrophin in skeletal muscles and evidence of contraction‐induced muscle injury from early life, leading to muscle degeneration and functional deterioration, which constitutes a fundamental phenotype of DMD (Burns et al., 2018; Burns, Drummond et al., 2019; Burns, Murphy et al., 2019; O'Halloran et al., 2023). Consequently, the dystrophin glycoprotein complex and its associated functionality are compromised, resulting in inflammation within skeletal muscle and the development of fibrosis. Functional respiratory muscle weakness is accompanied by diminished ventilatory capacity, ultimately leading to respiratory failure (De Bruin et al., 1997; Evans et al., 2009; Khirani et al., 2014; Smith et al., 1989). Currently, no cure exists for boys with DMD. Therefore, interventions aimed at enhancing respiratory muscle strength in DMD are important in attempting to delay respiratory muscle failure, thereby preserving respiratory system efficacy.

Damage and degeneration in human DMD starts early in the disease, with children exhibiting motor deficits at ∼2–3 years of age. The initial symptoms include challenges in climbing stairs, a distinctive waddling gait and recurrent falls (Emery, 2002). As the disease progresses rapidly, the muscle phenotype becomes more apparent. Patients as early as 9 years of age are wheelchair dependent; they experience loss of upper limb function and respiratory insufficiency, which requires assisted ventilation by 20 years of age (Emery, 2002). Improvements in the standards of clinical care, such as non‐invasive support with mechanical ventilation, ongoing management of deformities in the spine and earlier implementation of therapies, have translated into the extension of median life expectancy for patients with DMD from late teens to late twenties (Broomfield et al., 2021; Passamano et al., 2012).

One of the most widely studied models of DMD is the dystrophin‐deficient *mdx* mouse. Studies by our group have illustrated compromised respiratory muscle function in the *mdx* mouse, which models the human DMD condition. Specifically, investigations into the respiratory system of the *mdx* mouse model have revealed pronounced muscle weakness alongside structural remodelling of the diaphragm, the primary muscle of breathing (Burns et al., 2018, 2017; Burns, Drummond et al., 2019; Burns, Murphy et al., 2019; Mhandire et al., 2022; O'Halloran et al., 2023). Inflammatory markers, such as immune cell infiltration, increased cytokine concentrations, elevated levels of reactive oxygen species and collagen deposition, are evident in the *mdx* diaphragm (Burns, Drummond et al., 2019; Choi et al., 2016; O'Halloran et al., 2023). EMG activity of the obligatory muscles of breathing in the *mdx* model of DMD is reduced, which most probably reflects impaired neuromuscular function pertinent to respiratory performance (Burns, Murphy et al., 2019; O'Halloran et al., 2023; Personius & Sawyer, 2006).

Glucocorticoids, such as prednisone/prednisolone or deflazacort, are the standard treatment of care for boys with DMD, which aims to offset the progression of the disease by promoting anti‐inflammatory pathways. Although glucocorticoids have benefits, their chronic use is also associated with adverse side‐effects that can decrease quality of life, including muscle fibre atrophy, decreased muscle force, weight gain and bone loss. Interestingly, patients can also develop resistance to glucocorticoids. Numerous strategies are being explored in preclinical investigations to mitigate these adverse effects, with the aim of translation to humans. Quattrocelli, Barefield et al. (2017) demonstrated that administering glucocorticoids on a weekly basis, as opposed to a daily regimen, for 4 weeks, is effective in diminishing the harmful consequences associated with chronic glucocorticoid treatment in the D2.*mdx* mouse model of DMD. Their findings indicated that weekly glucocorticoid treatment resulted in improved functional outcomes, including enhanced grip strength, extended endurance and increased tetanic force in the tibialis anterior muscle (Quattrocelli, Barefield et al., 2017). Additionally, the weekly regimen was associated with an increase in the cross‐sectional areas of the gastrocnemius and diaphragm muscles, whereas these measures were found to be reduced following chronic daily administration, indicating that daily steroid dosing elicits an atrophic effect (Quattrocelli, Barefield et al., 2017).

The antioxidant *N*‐acetylcysteine (NAC) has been shown to exert anti‐inflammatory and anti‐fibrotic effects (Tenório et al., 2021), improving respiratory muscle performance in a range of animal models of muscle dysfunction, including *mdx* mice (Burns, Drummond et al., 2019; Redwan et al., 2023; Terrill et al., 2012). Although 1% NAC was ineffective in supporting respiratory system performance in *mdx* mice (Maxwell et al., 2024), which was disappointing and surprising based upon our previous findings over a shorter time frame of 2 weeks of treatment (Burns, Drummond et al., 2019) and prior findings of a range of improvements in *mdx* limb muscle, including increased force generation (Pinniger et al., 2017) also after 2 weeks of treatment, we nevertheless sought value in considering the potential benefit of co‐administration of NAC and PRED. We reasoned that combined treatment might be superior considering the potential for PRED administered chronically (albeit weekly for 3 months in this study) to drive adverse outcomes for muscle per se and active respiratory muscles in particular, since chronic PRED treatment can lead to muscle wasting (Danon & Carpenter, 1991; Quattrocelli, Barefield et al., 2017). We have previously shown that NAC prevents respiratory muscle atrophy and weakness in chronically hypoxic mice (Lewis et al., 2016) and prevents diaphragm dysfunction in rats exposed to chronic intermittent hypoxia (Shortt et al., 2014). Therefore, NAC has the capacity to prevent stress‐induced muscle wasting and weakness, and although NAC alone does not improve *mdx* diaphragm function, combination therapy (PREDNAC) could conceivably be superior to PRED in improving *mdx* diaphragm function, acknowledging too that this is speculative.

In the present study, we sought to assess the effects of PRED once weekly and a combination therapy of PRED and NAC (PREDNAC) on respiratory system performance in the *mdx* mouse model of DMD starting at 1 month of age for a duration of 3 months. We hypothesized that both PRED treatment and the combination therapy (PREDNAC) would be beneficial to *mdx* diaphragm muscle form, improve *mdx* diaphragm force generation and rescue impaired *mdx* respiratory EMG activity (O'Halloran et al., 2023).

## 2 MATERIALS AND METHODS

### 2.1 Ethical approval

Procedures on live animals were performed under project authorization (AE19130/P117) from the Health Products Regulatory Authority in accordance with Irish and European law, with prior ethical approval by University College Cork (AEEC 2019/013). Experiments were carried out in accordance with guidelines and requirements laid down by University College Cork's Animal Welfare Body. We adhered to *Experimental Physiology*’s policies regarding animal experiments.

### 2.2 Experimental animals, PRED and PREDNAC treatment

**FIGURE 1:**
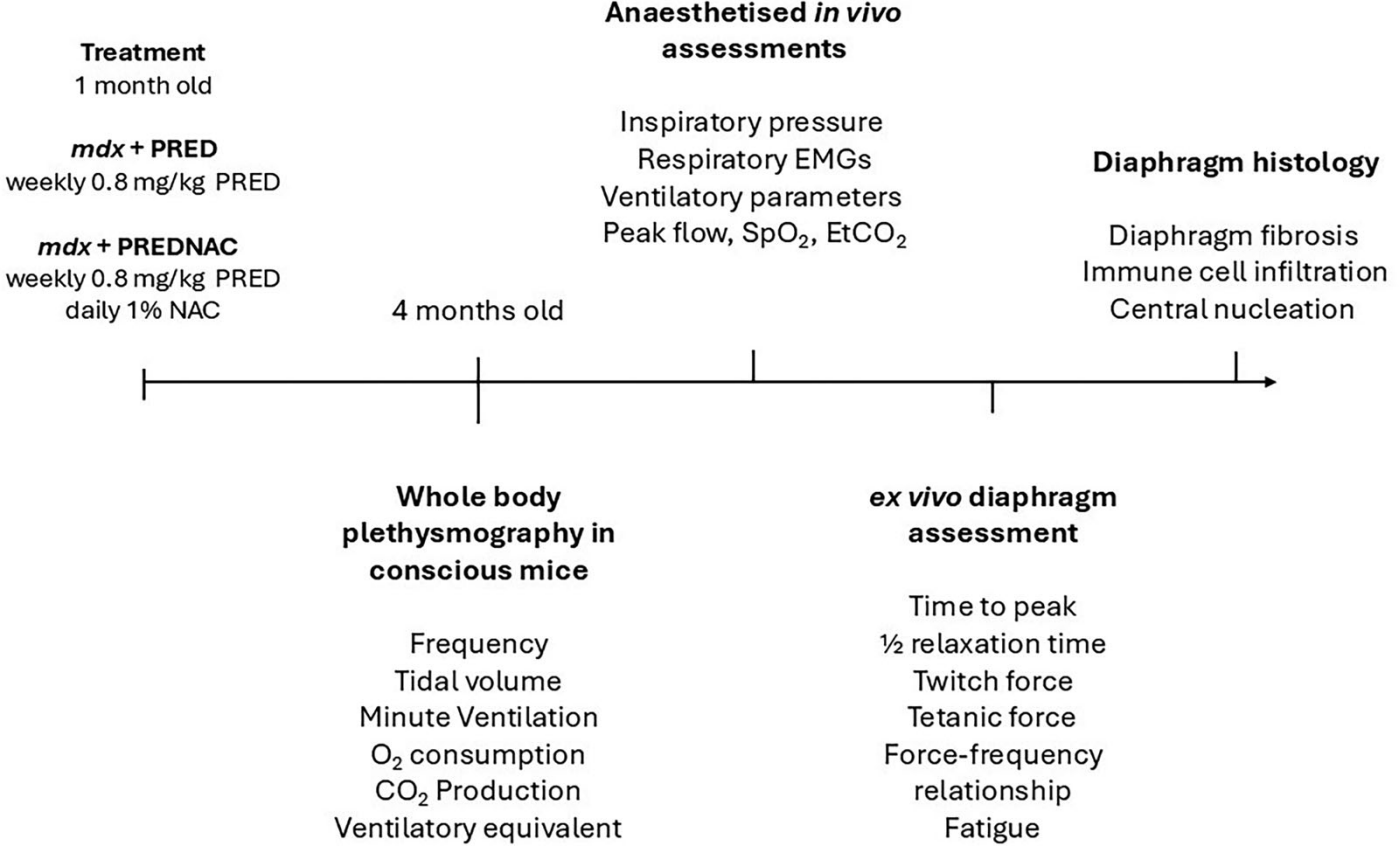
Schematic diagram of the time line of the experimental protocol, experimental groups and parameters measured. Abbreviations: EtCO_2_, end‐tidal CO_2_; PRED, α‐methylprednisolone; PREDNAC, α‐methylprednisolone plus *N*‐acetyl cysteine; $S_{pO_{2}}$, peripheral capillary O_2_ saturation.

Breeding pairs for wild‐type (C57BL/10ScSnJ) and *mdx* mice (C57BL/10ScSn‐Dmd*mdx*/J) were purchased from the Jackson Laboratory (Bar Harbor, ME, USA), and colonies were established at University College Cork's specific pathogen‐free facility. Animals were group housed in individually ventilated cages in temperature‐ and humidity‐controlled rooms, operating on a 12 h light–12 h dark cycle, with food and water available ad libitum. Studies were performed on 60 male mice. Wild‐type mice were randomly selected from our colony (*n* = 15); *mdx* mice were randomly assigned to one of three groups: *mdx* (*n* = 15); *mdx* + PRED (*n* = 15); and *mdx* + PREDNAC (*n* = 15). A schematic time line of the study protocol is shown in Figure 1.

The *mdx* + PRED group received 0.8 mg/kg of α‐methylprednisolone once weekly i.p. for 3 months, beginning at 1 month of age. The *mdx* + PREDNAC group received 0.8 mg/kg of α‐methylprednisolone once weekly i.p. and 1% *N*‐acetyl cysteine daily (Sigma‐Aldrich, Wicklow, Ireland) in the drinking water for 3 months, beginning at 1 month of age.

α‐Methylprednisolone was prepared fresh on the day of injection. Drinking water containing NAC was pH matched to control water and prepared fresh each day. For both treatment groups, fluid intake was estimated by weighing water bottles daily and estimating fluid intake per mouse. Fluid intake was normal and stable over the 3 month intervention, averaging 4–5 mL per mouse per day. Mice were studied at 4 months of age. A thorough assessment of respiratory performance was made, with measurements of breathing and ventilatory capacity in response to chemo‐activation in conscious animals, recordings of thoracic inspiratory pressure and respiratory muscle EMG in anaesthetized animals, and respiratory muscle function tests *ex vivo*. Diaphragm muscle tissue was collected for structural analysis using standard histological techniques.

### 2.3 Plethysmography

Whole‐body plethysmography was used to assess respiratory flow in unrestrained, conscious wild‐type (*n* = 13), *mdx* (*n* = 13), *mdx* + PRED (*n* = 12) and *mdx* + PREDNAC (*n* = 12) mice. Mice were introduced into plethysmography chambers (model PLY4211; volume 600 mL; Buxco Research Systems, Wilmington, NC, USA) and were allowed an acclimation period (∼30 min) with room air passing through each chamber (0.85 L/min).

#### 2.3.1 Experimental protocol

Following acclimation, combined central and peripheral chemoreceptor stimulation with hypercapnic hypoxia (HcHx; 10% O_2_ and 6% CO_2_) was performed for 10 min, to examine maximum chemoactivated breathing. Following a 20 min wash‐out period, during confirmed periods of quiet rest, a 10 min baseline recording was performed in room air to measure normal tidal breathing. Respiratory parameters, including respiratory frequency (*f*), tidal volume (*V*_T_) and minute ventilation (${\dot{V}}_{I}$), were recorded on a breath‐by‐breath basis for analysis offline. A gas analyser (ADInstruments, Colorado Springs, CO, USA) was used to determine oxygen consumption (${\dot{V}}_{O_{2}}$) and carbon dioxide production (${\dot{V}}_{CO_{2}}$).

#### 2.3.2 Data analysis

Baseline normoxic ventilation was determined as an average of the 10 min baseline period during a continuous period of confirmed quiet rest. For HcHx, ventilatory measurements were taken during the final 5 min of the challenge to ensure steady‐state changes. The *V*_T_ and ${\dot{V}}_{I}$ were normalized for body mass (in grams). The ventilatory equivalents for oxygen (V˙I/V˙IV˙O2V˙O2) and carbon dioxide (V˙I/V˙IV˙CO2V˙CO2) were determined.

### 2.4 Respiratory EMGs and inspiratory pressure recordings

Following plethysmography measurements and with additional animals [wild‐type (*n* = 15), *mdx* (*n* = 15), *mdx* + PRED (*n* = 13) and *mdx* + PREDNAC (*n* = 14) mice], anaesthesia was induced with 5% isoflurane (Vetflurane, VIRBAC Ltd., Suffolk, UK) in 60% O_2_ (balance N_2_) in an induction chamber. Mice were subsequently placed in the supine position and received 2% isoflurane in 60% O_2_ (balance N_2_) by nose‐cone delivery. Mice were gradually transitioned from isoflurane to urethane anaesthesia (1.7 g/kg i.p. in total given in three injections) over a 25 min period. Urethane was purchased from Sigma‐Aldrich, Wicklow, Ireland. Body temperature was maintained at 37°C–38°C via a rectal probe and thermostatically controlled heating blanket (Harvard Apparatus, Holliston, MA, USA). We established an acceptable surgical plane of anaesthesia, determined by an absent pedal withdrawal reflex and no somatic motor response to noxious pinch. Supplemental anaesthetic was administered as required. A pulse oximeter clip (MouseOx™; Starr Life Sciences Corporation, Oakmount, PA, USA) was placed on a shaved thigh for the measurement of peripheral capillary O_2_ saturation ($S_{pO_{2}}$). A mid‐cervical tracheotomy was performed. All animals were maintained with a bias flow of supplemental O_2_ [fraction of inspired oxygen ($F_{I,O_{2}}$) = 0.60] in baseline conditions. Oesophageal pressure was measured using a pressure‐tip catheter (Mikro‐Tip; Millar Inc., Houston, TX, USA), which was positioned in the thoracic oesophagus through the mouth. The catheter was advanced into the stomach to record positive pressure swings during inspiration, then withdrawn into the lower oesophagus, where stable phasic sub‐atmospheric pressure swings during inspiration were observed. Concentric needle monopolar recording electrodes (26 gauge; Natus Manufacturing Ltd, Ireland) were inserted into the middle costal region of the diaphragm on the right‐hand side for the continuous measurement of diaphragm EMG activity, into an external intercostal, in the second to fourth rostroventral intercostal space, for the measurement of external intercostal EMG, and into the parasternal intercostal (second or third space) for the measurement of parasternal intercostal EMG. In addition, concentric needle monopolar electrodes were used to record scalene, cleidomastoid, sternomastoid, sternohyoid (all mid‐belly insertions) and trapezius (superficial insertion in the upper region on either side) with contemporaneous recordings of eight EMG signals in each mouse. EMG signals were amplified (×5000), filtered (500 Hz low cut‐off to 5000 Hz high cut‐off) and integrated (50 ms time constant; Neurolog system; Digitimer Ltd, UK). All signals were passed through an analog‐to‐digital converter (Powerlab r8/30; ADInstruments, Colorado Springs, CO, USA) and were acquired using LabChart 8 (ADInstruments) sampled at 20 kHz (EMG) and 1 kHz for other parameters (tracheal airflow and pressure, and $S_{pO_{2}}$).

#### 2.4.1 Experimental protocol

Following instrumentation, animals were allowed to stabilize before baseline parameters were measured. Next, animals were challenged with a single sustained tracheal occlusion until peak inspiratory efforts (identified as stable, maximal successive efforts, often until task failure) were observed in the inspiratory pressure recordings during sustained maximum non‐ventilatory efforts. Following recovery, animals were instrumented for the measurement of tracheal airflow, and parameters were recorded during a newly established second baseline (prevagotomy) period. Subsequently, the vagi were sectioned bilaterally at the cervical level. Respiratory parameters were recorded in steady‐state conditions for a minimum of 10 min following vagotomy. Next, animals were challenged with HcHx [$F_{I,O_{2}}$ = 0.15 and fraction of inspired carbon dioxide ($F_{I,CO_{2}}$) = 0.06; 2 min] to examine the effects of chemostimulation on the diaphragm, external intercostal, parasternal intercostal, cleidomastoid, sternomastoid, sternohyoid, scalene and trapezius EMG activities and ventilatory parameters. After the experimental protocol, anaesthetized mice were killed by cervical dislocation, and death was confirmed by the absence of cardiac rhythm.

#### 2.4.2 Data analysis

The amplitudes of integrated inspiratory diaphragm, external intercostal, parasternal intercostal, cleidomastoid, sternomastoid, sternohyoid, scalene and trapezius EMG activities and peak inspiratory sub‐atmospheric oesophageal pressure change from baseline were measured and averaged in steady‐state basal conditions (typically over 1 min) and averaged for the five successive maximal sustained efforts (maximal response) of the single airway occlusion challenge (Burns, Murphy et al., 2019). During baseline breathing and chemo‐activation, peak inspiratory and expiratory flows were measured, and inspiratory tidal volume was derived from the integral of tracheal airflow measurements.

### 2.5 *Ex vivo* diaphragm muscle function

Following in vivo pressure and EMG measurements mice were killed by cervical dislocation, and the diaphragm muscle (rib and central tendon intact) was immediately excised and placed in a tissue bath at room temperature containing continuously gassed hyperoxic (95% O_2_–5% CO_2_) Krebs solution (mM: NaCl, 120; KCl, 5; calcium gluconate, 2.5; MgSO_4_, 1.2; NaH_2_PO_4_, 1.2; NaHCO_3_, 25; and glucose, 11.5) and *d*‐tubocurarine (25 µM) prior to functional analysis. Thin strips of diaphragm muscle were prepared from the mid‐costal section of the right hemidiaphragm. Diaphragm muscle preparations were suspended vertically between two platinum plate electrodes in a water‐jacketed tissue bath at 35°C containing Krebs solution and were continuously gassed with carbogen (95% O_2_–5% CO_2_). The rib was sutured to an immobile hook and remained in a fixed position for the duration of the experiment. Using non‐elastic string, the central tendon was attached to a lever connected to a dual‐mode force transducer (Aurora Scientific Inc., Aurora, ON, Canada). To determine muscle optimum length (*L*_o_), the length of the muscle preparations was adjusted using a micro‐positioner between intermittent twitch contractions. The muscle length that revealed maximal isometric twitch force for a single isometric twitch stimulation (supramaximal stimulation, 1 ms duration) was considered *L*_o_. Diaphragm preparations were maintained at *L*_o_ for the duration of the protocol.

#### 2.5.1 Experimental protocol

First, peak isometric twitch force was measured via stimulation of the muscle preparation at 100 Hz for 1 ms duration. Time‐to‐peak force and half‐relaxation time parameters were derived from resultant twitch contractions. Isometric tetanic force was assessed via stimulation of the muscle preparation at 100 Hz for 300 ms duration. To examine the force–frequency relationship, the muscle preparation was stimulated sequentially at 25, 50, 75, 100, 125 and 150 Hz (300 ms train duration), interspersed by 1 min intervals. Finally, a fatiguing protocol was run involving 150 sequential submaximal contractions at 40 Hz, separated by 2 s intervals.

#### 2.5.2 Data analysis

Muscle bundle cross‐sectional area was determined for the purpose of normalizing muscle force to bundle size. Cross‐sectional area was calculated by dividing muscle mass (weight, in grams) by the product of muscle *L*_o_ (in centimetres) and muscle density (assumed to be 1.06 g/cm^3^). Muscle force was divided by bundle cross‐sectional area and expressed as a specific force (in newtons per centimetre squared). Time‐to‐peak force and half‐relaxation time were measured as indices of isometric twitch kinetics and were expressed in milliseconds.

### 2.6 Muscle histology

#### 2.6.1 Tissue preparation

Sections of hemidiaphragm from wild‐type (*n* = 10), *mdx* (*n* = 10), *mdx* + PRED (*n* = 10) and *mdx* + PREDNAC (*n* = 10) mice were mounted on cubes of liver. Diaphragm samples were embedded in optimum cutting temperature embedding medium (OCT; VWR International, Dublin, Ireland) for cryoprotection, then frozen in isopentane (Sigma–Aldrich, Wicklow, Ireland) cooled on dry ice. Samples were then stored at −80°C for subsequent structural analysis. Serial transverse muscle sections (10 µm thick) were cut using a cryostat (Leica CM1950; Leica Microsystems, Nussloch, Germany) at −20°C and mounted across polylysine‐coated glass slides (VWR International, Dublin, Ireland) allowing for a distribution of tissue on a given slide.

#### 2.6.2 Histological analysis

To examine putative inflammatory cell infiltration and the relative density of centrally nucleated myofibres in diaphragm, tissue sections were stained with Haematoxylin and Eosin using an autostainer (Leica ST5010 Autostainer XL; Leica Microsystems, Nussloch, Germany). Slides were mounted using DPX mounting medium (Sigma–Aldrich, Wicklow, Ireland), air‐dried and visualized on a bright field microscope (Olympus BX51) at ×20 magnification.

To determine the relative area of collagen deposition, Picrosirius Red (Leica Biosystems, Wetzlar, Germany) staining was completed. Slides were mounted using DPX mounting medium (Sigma–Aldrich, Wicklow, Ireland), air‐dried and visualized on a bright field microscope (Olympus BX51) at ×10 magnification.

#### 2.6.3 Data analysis

A total of four tissue sections per animal were examined. Muscle histology was scored using ImageJ software. Putative inflammatory cell infiltration (the presence of nucleated cells in the extracellular matrix) was scored and expressed as a percentage of the total area of muscle within the area randomly selected. The number of centrally nucleated cells (the presence of cells where the nuclei are located centrally and not peripherally in the muscle cell) was scored and expressed as a percentage of the total muscle cells within the area randomly selected. For slides stained with Picrosirius Red, the microscope lighting exposure was standardized during imaging. Images were analysed using a colour balance threshold, and the area of collagen was expressed as a percentage of the total area of muscle. Data generated from multiple images with varying regions of interest per muscle were averaged per animal before computing group means.

### 2.7 Statistical analysis

Values are expressed as box and whisker plots (median, interquartile range and individual data scatter plot) in graphs. One‐way ANOVA was used statistically to compare body mass, inflammatory cell infiltrate, collagen deposition, diaphragm muscle contractile function and respiratory parameters (Table 1). All other data were compared statistically by two‐way ANOVA (or mixed model when occasional data points were missing for technical reasons) with Tukey's multiple comparisons *post hoc* test (Prism v.10.3.1). Values of *p* < 0.05 are reported, and *p* < 0.05 was considered statistically significant.

**TABLE 1:** Respiratory parameters during baseline conditions, following bilateral vagotomy and during exposure to hypercapnic hypoxia in anaesthetized wild‐type, *mdx*, *mdx* + PRED and *mdx* + PREDNAC groups of mice.

Parameter	Wild‐type (*n* = 15)	*mdx* (*n* = 15)	*mdx* + PRED (*n* = 13)	*mdx* + PREDNAC (*n* = 14)	ANOVA *p*‐value
Baseline					
Respiratory frequency (breaths/min)	187 ± 31	217 ± 37	220 ± 34	209 ± 45	0.0771
Tidal volume (µL/g)	4.4 ± 0.7	5.1 ± 0.8	5.1 ± 1.4	4.1 ± 1.1	**0.0400**
Minute ventilation (mL/g/min)	0.8 ± 0.2	1.1 ± 0.2	1.1 ± 0.3	0.9 ± 0.3	**0.0123**
Peak inspiratory flow (mL/s)	2.0 ± 0.3	2.7 ± 0.4	2.7 ± 0.3	2.6 ± 0.5	**0.0005**
Peak expiratory flow (mL/s)	4.2 ± 0.6	5.3 ± 0.5	5.3 ± 1.0	5.1 ± 1.0	**0.0090**
$S_{pO_{2}}$ (%)	96.3 ± 3.3	96.6 ± 1.8	96.7 ± 1.0	96.9 ± 0.7	0.6540
End‐tidal CO_2_ (%)	8.2 ± 1.2	8.5 ± 0.7	8.5 ± 1.2	9.1 ± 0.9	0.1206
Vagotomy					
Respiratory frequency (breaths/min)	49 ± 12	72 ± 16	61 ± 13	62 ± 9	**0.0005**
Tidal volume (µL/g)	13.9 ± 6.2	11.7 ± 5.3	13.9 ± 9.0	8.9 ± 1.2	0.0973
Minute ventilation (mL/g/min)	0.7 ± 0.3	0.8 ± 0.2	0.8 ± 0.5	0.6 ± 0.1	0.1000
Peak inspiratory flow (mL/s)	6.8 ± 1.5	5.9 ± 2.2	6.5 ± 2.6	5.8 ± 1.8	0.4084
Peak expiratory flow (mL/s)	8.1 ± 1.3	7.7 ± 1.9	7.8 ± 1.3	8.4 ± 1.8	0.5411
$S_{pO_{2}}$ (%)	97.5 ± 1.0	97.1 ± 0.5	96.8 ± 0.9	97.1 ± 0.5	0.1764
End‐tidal CO_2_ (%)	7.5 ± 1.2	8.1 ± 1.0	7.9 ± 1.5	8.9 ± 1.2	0.1462
Hypercapnic hypoxia					
Respiratory frequency (breaths/min)	39 ± 10	53 ± 16	43 ± 15	41 ± 10	**0.0319**
Tidal volume (µL/g)	19.2 ± 7.5	19.1 ± 7.3	22.3 ± 12.8	16.0 ± 3.2	0.2496
Minute ventilation (mL/g/min)	0.7 ± 0.2	0.9 ± 0.3	0.9 ± 0.5	0.6 ± 0.1	**0.0209**
Peak inspiratory flow (mL/s)	8.6 ± 2.6	7.5 ± 2.4	5.8 ± 1.8	8.4 ± 2.3	0.3232
Peak expiratory flow (mL/s)	8.6 ± 1.1	8.7 ± 1.2	9.8 ± 1.3	8.8 ± 1.1	0.0561
$S_{pO_{2}}$ (%)	50.9 ± 9.8	52.3 ± 8.5	54.6 ± 7.6	51.2 ± 6.4	0.6609
End‐tidal CO_2_ (%)	>10	>10	>10	>10	

*Notes*: Values are expressed as means ± SD and were compared statistically by one‐way ANOVA (or mixed model when occasional data points were missing for technical reasons) with Tukey's multiple comparisons *post hoc* test (Prism v.10.3.1). Exact *p*‐values are reported for all comparisons. Significant differences are highlighted by *p*‐values in bold.

Abbreviation: $S_{pO_{2}}$, peripheral capillary O_2_ saturation.

## 3 RESULTS

### 3.1 Effect of PRED and PREDNAC on body mass

**FIGURE 2:**
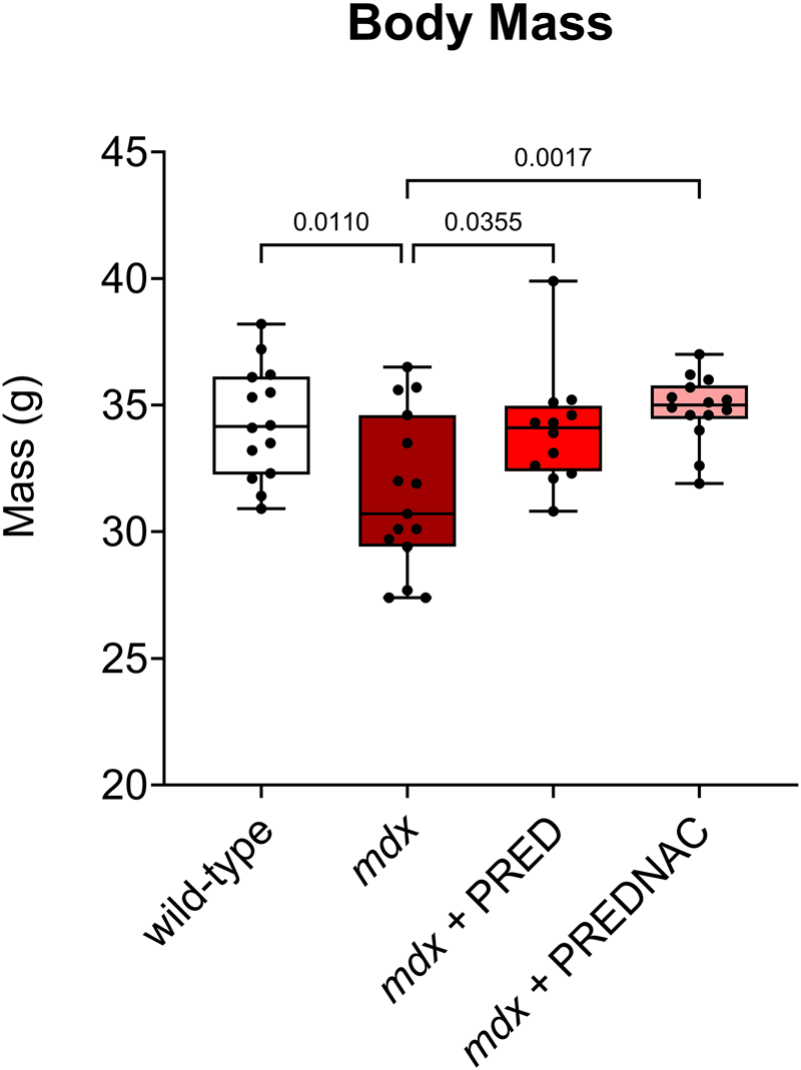
Body mass in wild‐type (*n* = 14), *mdx* (*n* = 15), *mdx* + PRED (*n* = 12) and *mdx* +PREDNAC (*n* = 14) mice. Values are expressed as box and whisker plots (median, 25th–75th percentile and scatter plot) and were compared statistically using one‐way ANOVA with Tukey's *post hoc* tests. Values of *p* < 0.05 are reported. Abbreviations: PRED, α‐methylprednisolone; PREDNAC, α‐methylprednisolone plus *N*‐acetyl cysteine.

Body mass was reduced in *mdx* mice compared with wild‐type control mice (*p* = 0.011). Both PRED (*p* = 0.0355) and PREDNAC (*p* = 0.0017) treatments recovered body mass in the *mdx* mouse model of DMD such that it was equivalent to wild type (Figure 2).

### 3.2 Baseline ventilation and ventilatory responsiveness to hypercapnic hypoxia in conscious mice

**FIGURE 3:**
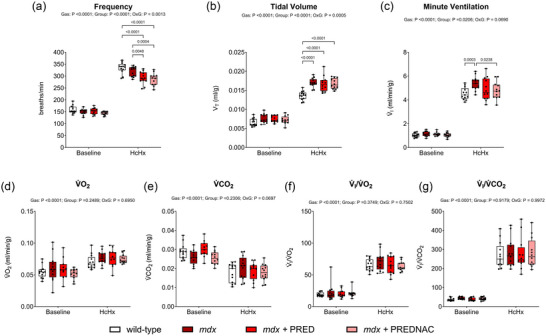
Assessment of breathing and metabolism during baseline conditions and exposure to hypercapnic hypoxia in conscious wild‐type (*n* = 13), *mdx* (*n* = 13), *mdx* + PRED (*n* = 12) and *mdx* + PREDNAC (*n* = 12) mice. (a–c) Frequency (a), tidal volume (b) and minute ventilation (c) were determined from measurement of respiratory airflow. (d–g) The oxygen consumption (d) and carbon dioxide production (e) were measured, and the ventilatory equivalent for oxygen (V˙I/V˙IV˙O2V˙O2; f) and for carbon dioxide (V˙I/V˙IV˙CO2V˙CO2; g) were determined offline. Data were compared statistically by two‐way ANOVA (or mixed model when occasional data points were missing for technical reasons) with Tukey's multiple comparisons *post hoc* test (Prism v.10.3.1). Values of *p* < 0.05 are reported. Abbreviations: HcHx, hypercapnic hypoxia; PRED, α‐methylprednisolone; PREDNAC, α‐methylprednisolone plus *N*‐acetyl cysteine; ${\dot{V}}_{CO_{2}}$, carbon dioxide production; ${\dot{V}}_{O_{2}}$, oxygen consumption.

Respiratory frequency, *V*_T_, ${\dot{V}}_{I}$, metabolic ${\dot{V}}_{O_{2}}$, metabolic ${\dot{V}}_{CO_{2}}$ and the V˙I/V˙IV˙O2V˙O2 and V˙I/V˙IV˙CO2V˙CO2 during baseline conditions ($F_{I,O_{2}}$ = 0.209) and HcHx ($F_{I,O_{2}}$ = 0.10 and $F_{I,CO_{2}}$ = 0.06) are illustrated in Figure 3. Baseline values were similar between groups across all parameters measured. In response to HcHx, tidal volume (*p *< 0.0001) and minute ventilation (*p* = 0.0003) increased in *mdx* mice to a greater extent than wild‐type mice, but no differences were evident between wild‐type and *mdx* mice in V˙I/V˙IV˙O2V˙O2 and V˙I/V˙IV˙CO2V˙CO2. Neither chronic PRED nor PREDNAC treatment influenced integrated ventilatory performance in the *mdx* mouse model of DMD.

### 3.3 Baseline and peak inspiratory pressure and EMG activity in anaesthetized mice

**FIGURE 4:**
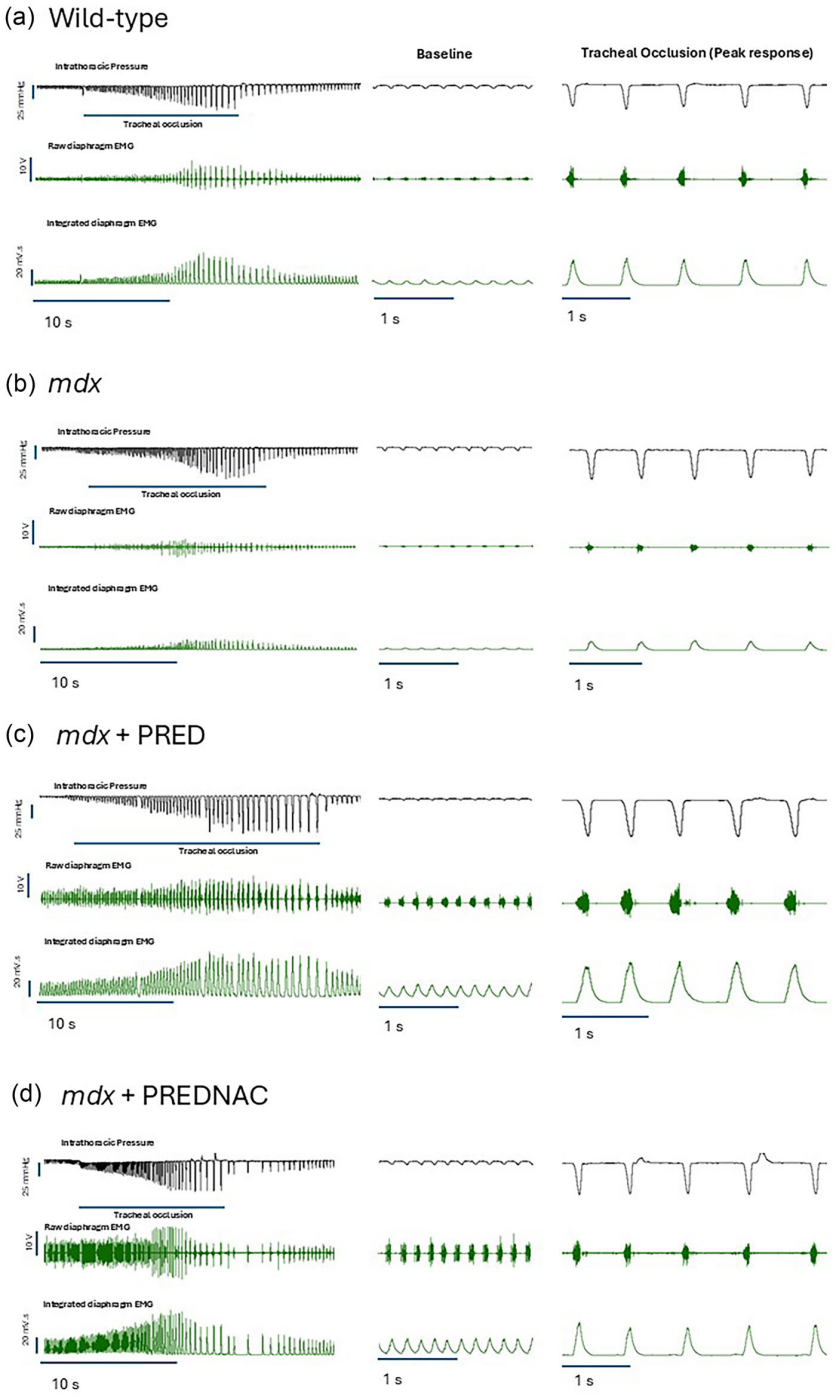
Original traces of intrathoracic pressure and diaphragm raw and integrated EMG activities in anaesthetized wild‐type (a), *mdx* (b), *mdx* + PRED (c) and *mdx* + PREDNAC (d) mice during baseline conditions and during tracheal occlusion evoking maximum EMG and peak pressure responses. The left panels show the entire trial. The middle panels show a portion of the baseline immediately preceding the airway occlusion. The right panels show the five successive peak responses during the tracheal occlusion. Abbreviations: PRED, α‐methylprednisolone; PREDNAC, α‐methylprednisolone plus *N*‐acetyl cysteine.

Representative original traces of inspiratory pressure and raw and integrated EMG activity before and during the tracheal occlusion manoeuvre are presented in Figure 4 (left‐hand panel). Representative original traces of baseline conditions and the five successive maximum efforts (peak response) in wild‐type (Figure 4a), *mdx* (Figure 4b), *mdx* + PRED (Figure 4c) and *mdx* + PREDNAC (Figure 4d) mice are presented in Figure 4 (middle and right‐hand panels).

**FIGURE 5:**
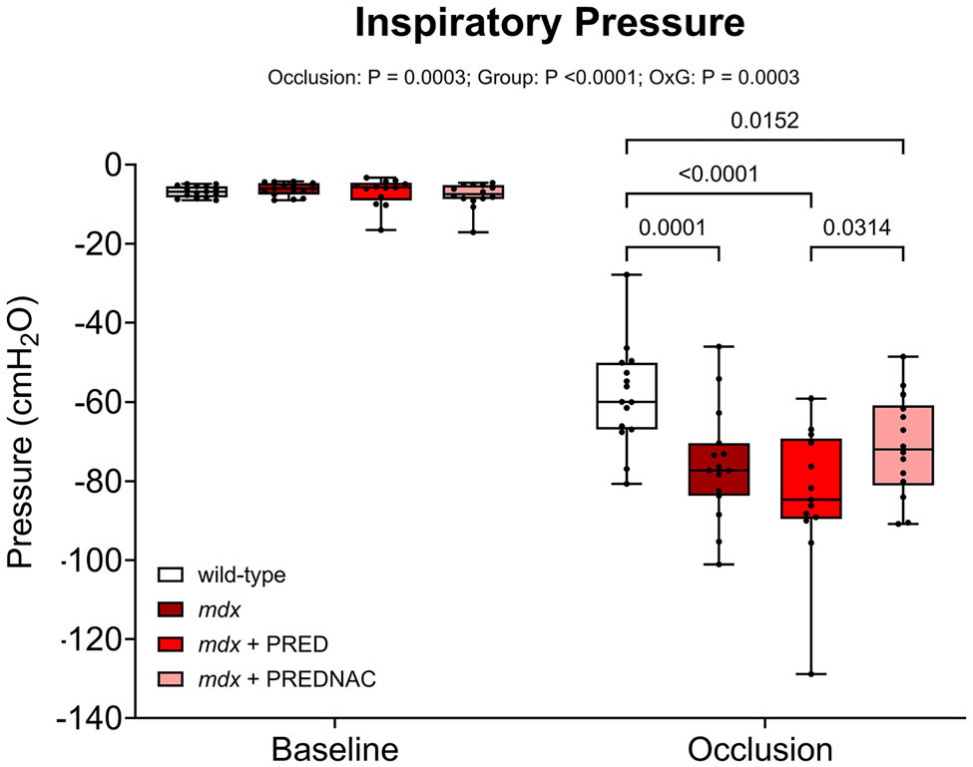
Inspiratory pressure generation during baseline and peak response during sustained tracheal occlusion in anaesthetized wild‐type (*n* = 15), *mdx* (*n* = 15), *mdx* + PRED (*n* = 13) and *mdx* + PREDNAC (*n* = 14) mice. Summary data showing inspiratory pressure‐generating capacity during baseline conditions and during tracheal occlusion (average of five successive greatest efforts). Values are expressed as box (median, 25th–75th percentile and individual data points) and whisker (minimum to maximum) plots. Data were compared statistically by two‐way ANOVA (or mixed model when occasional data points were missing for technical reasons) with Tukey's multiple comparisons *post hoc* test (Prism v.10.3.1). Values of *p* < 0.05 are reported. Abbreviations: PRED, α‐methylprednisolone; PREDNAC, α‐methylprednisolone plus *N*‐acetyl cysteine.

Summary data for inspiratory pressure‐generating capacity in baseline conditions and during protracted tracheal occlusion (peak response) are presented in Figure 5 for all groups. There were no differences in baseline inspiratory pressure generation across all groups. Inspiratory pressure was greater in *mdx* mice than in wild‐type control mice. A paradoxical increase in inspiratory pressure generation was observed in *mdx* mice at 4 months of age (*p* = 0.0001) owing to diaphragm remodelling and compensation afforded by accessory muscles of breathing (O'Halloran et al., 2023). Neither PRED nor PREDNAC influenced peak inspiratory capacity in the *mdx* mouse model of DMD.

**FIGURE 6:**
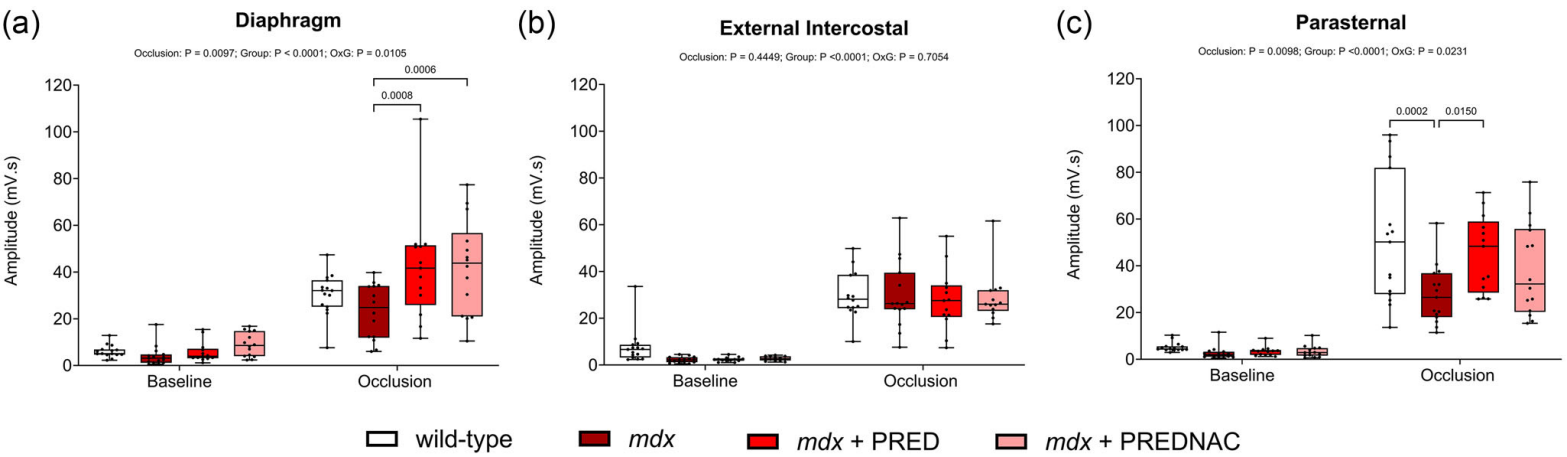
Diaphragm (a), external intercostal (b) and parasternal (c) respiratory EMG activities during baseline conditions and peak activation during tracheal occlusion in wild‐type (*n* = 15), *mdx* (*n* = 15), *mdx* + PRED (*n* = 13) and *mdx* + PREDNAC (*n* = 14) mice. Values are expressed as box (median, 25th–75th percentile and individual data points) and whisker (minimum to maximum) plots. Data were compared statistically by two‐way ANOVA (or mixed model when occasional data points were missing for technical reasons) with Tukey's multiple comparisons *post hoc* test (Prism v.10.3.1). Values of *p* < 0.05 are reported. Abbreviations: PRED, α‐methylprednisolone; PREDNAC, α‐methylprednisolone plus *N*‐acetyl cysteine.

Summary data of the obligatory inspiratory muscles (diaphragm, external intercostals and parasternal intercostals) during baseline conditions and peak responses during sustained tracheal occlusion are shown in Figure 6. There were no differences between groups in baseline conditions. There was a significant increase in diaphragm EMG activity in both drug treatment groups compared with *mdx* (*p* = 0.0008 for PRED and *p* = 0.0006 for PREDNAC). During peak activation, *mdx* parasternal EMG was significantly lower than wild‐type (*p* = 0.0002), and this was recovered with PRED treatment (*p* = 0.015).

**FIGURE 7:**
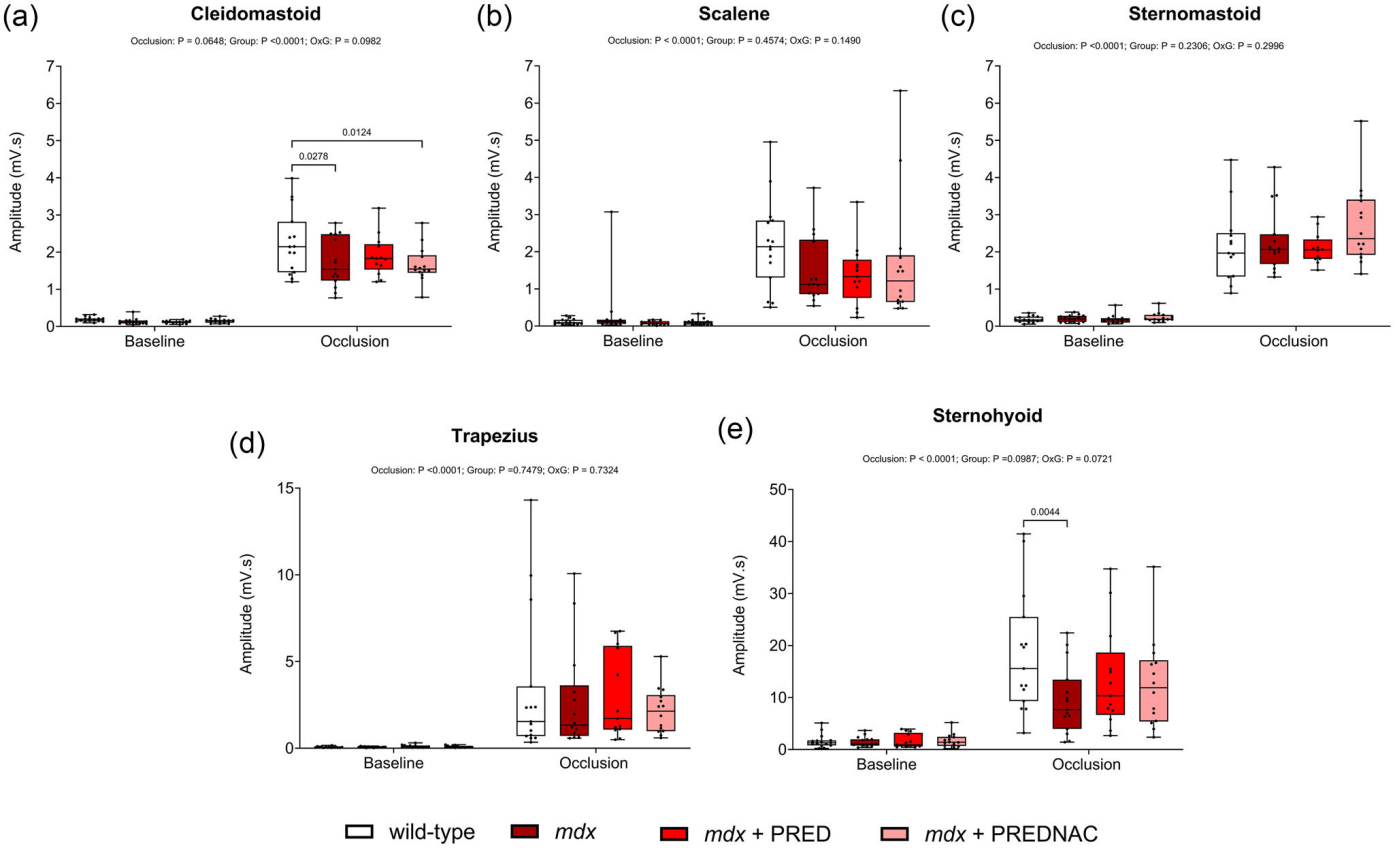
Cleidomastoid (a), scalene (b), sternomastoid (c), trapezius (d) and sternohyoid (e) respiratory EMG activity during baseline conditions and peak activation during tracheal occlusion in wild‐type (*n* = 15), *mdx* (*n* = 15), *mdx* + PRED (*n* = 13) and *mdx* + PREDNAC (*n* = 14) mice. Values are expressed as box (median, 25th–75th percentile and individual data points) and whisker (minimum to maximum) plots. Data were compared statistically by two‐way ANOVA (or mixed model when occasional data points were missing for technical reasons) with Tukey's multiple comparisons *post hoc* test (Prism v.10.3.1). Values of *p* < 0.05 are reported. Abbreviations: PRED, α‐methylprednisolone; PREDNAC, α‐methylprednisolone plus *N*‐acetyl cysteine.

Summary data for accessory muscle EMGs (cleidomastoid, scalene, sternomastoid, trapezius and sternohyoid) during baseline conditions and peak responses during sustained tracheal occlusion are presented in Figure 7. There were no differences between groups in baseline conditions. Cleidomastoid (*p* = 0.0278) and sternohyoid (*p* = 0.0044) EMG activities were lower in *mdx* compared with wild‐type mice. Drug treatments had no major effect on accessory EMG activities in *mdx* mice.

### 3.4 Respiratory parameters during baseline conditions, after vagotomy and during subsequent exposure to hypercapnic hypoxia in anaesthetized mice

**FIGURE 8:**
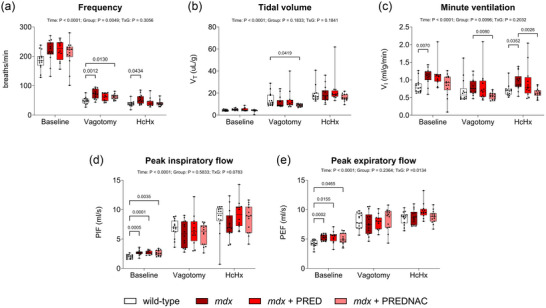
Summary data for respiratory frequency (a), tidal volume (b), minute ventilation (c), peak inspiratory flow (d) and peak expiratory flow (e) during baseline conditions, post‐vagotomy and during hypercapnic hypoxia exposure in anaesthetized wild‐type (*n* = 15), *mdx* (*n* = 15), *mdx* + PRED (*n* = 13) and *mdx* + PREDNAC (*n* = 14) mice. Values are expressed as box (median, 25th–75th percentile and individual data points) and whisker (minimum to maximum) plots. Data were compared statistically by two‐way ANOVA (or mixed model when occasional data points were missing for technical reasons) with Tukey's multiple comparisons *post hoc* test (Prism v.10.3.1). Values of *p* < 0.05 are reported. Abbreviations: *f*, respiratory frequency; HcHx, hypercapnic hypoxia; PEF, peak expiratory flow; PIF, peak inspiratory flow; PRED, α‐methylprednisolone; PREDNAC, α‐methylprednisolone plus *N*‐acetyl cysteine; ${\dot{V}}_{I}$, minute ventilation; *V*_T_, tidal volume.

**FIGURE 9:**
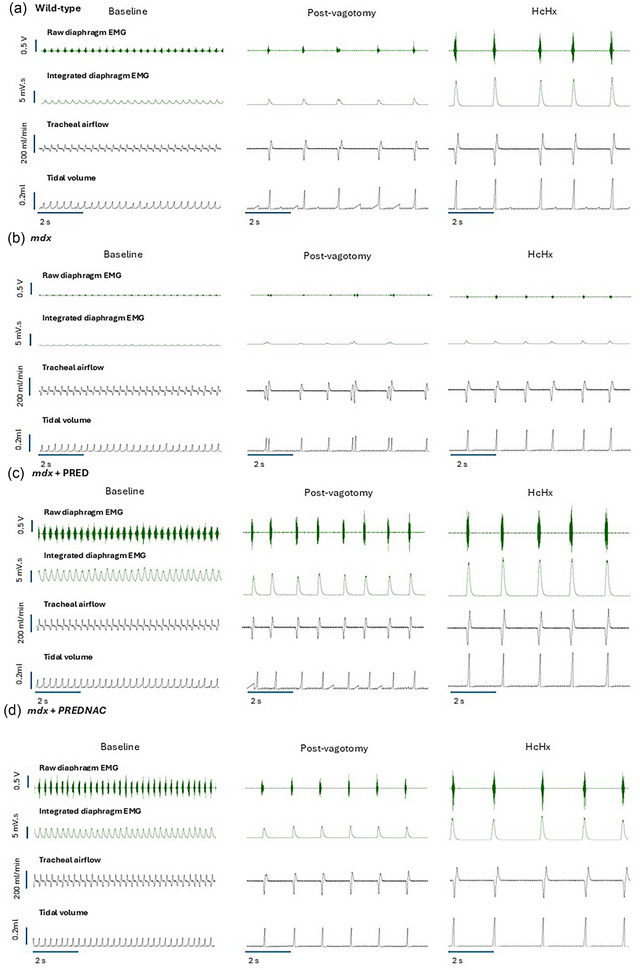
Original traces of respiratory parameters across the ventilatory range in anaesthetized wild‐type (a), *mdx* (b), *mdx* + PRED (c) and *mdx* + PREDNAC (d) mice. Diaphragm raw EMG activity, integrated diaphragm EMG activity, tracheal airflow and tidal volume in an anaesthetized *mdx* mouse during baseline conditions, following vagotomy and during hypercapnic hypoxia. Abbreviations: HcHx, hypercapnic hypoxia; PRED, α‐methylprednisolone; PREDNAC, α‐methylprednisolone plus *N*‐acetyl cysteine.

Group data for respiratory parameters across the ventilatory range are shown in Figure 8 and Table 1. Respiratory frequency and minute ventilation tended to be significantly higher in *mdx* compared with wild‐type mice. For the most part, drug treatments did not affect breathing parameters in *mdx* mice. Representative original traces of diaphragm raw and integrated EMG activity, tracheal airflow and tidal volume are presented in Figure 9 for all groups across a range of behaviours: baseline conditions, following bilateral vagotomy (increased central respiratory drive) and during exposure to HcHx (a further boost to central respiratory drive).

### 3.5 Inflammatory cell infiltration and collagen deposition in diaphragm muscle

**FIGURE 10:**
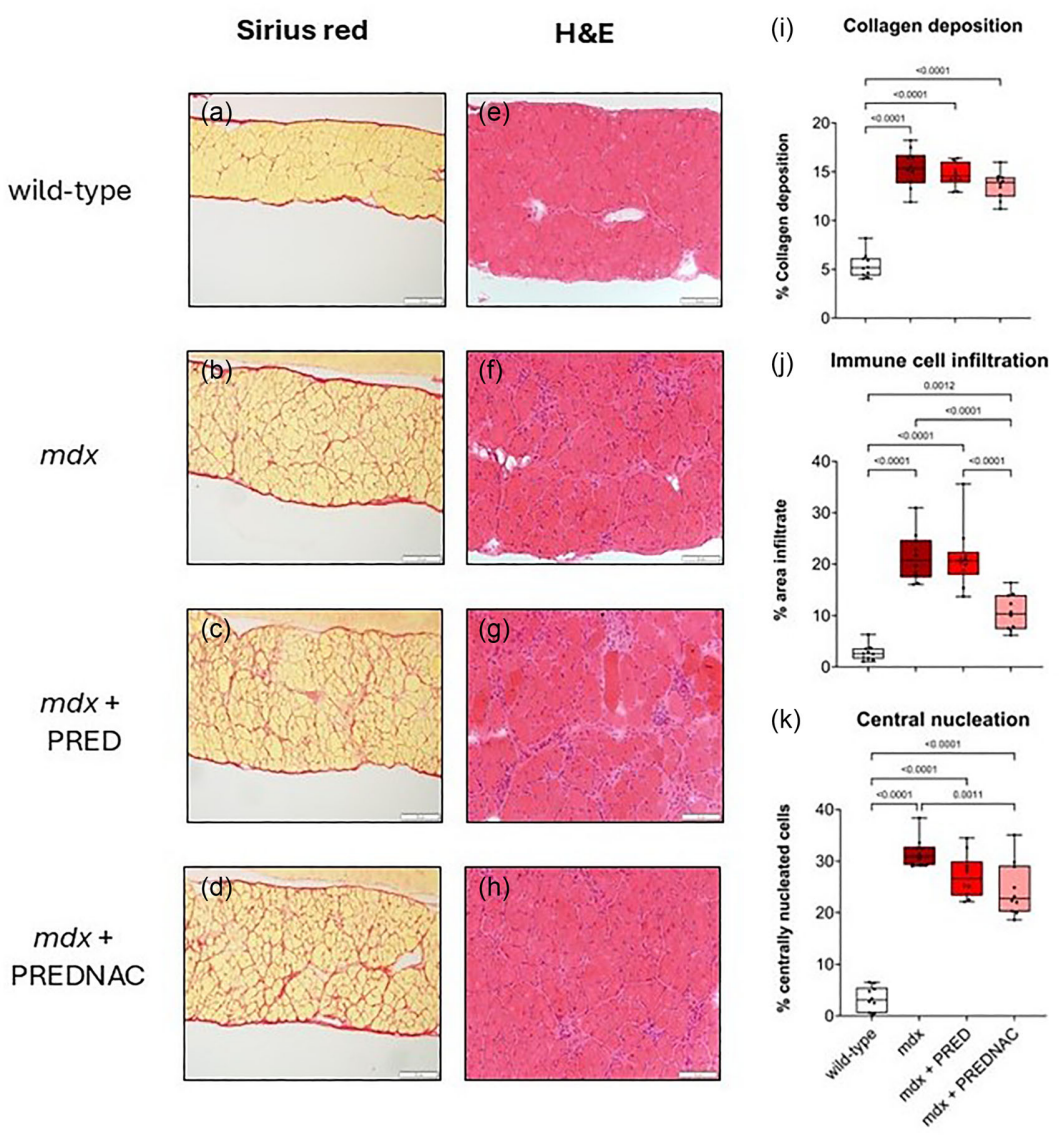
Assessment of collagen deposition and immune cell infiltration in diaphragm muscle from wild‐type (*n* = 10), *mdx* (*n* = 10), *mdx* + PRED (*n* = 10) and *mdx* + PREDNAC (*n* = 10) mice. (a–h) Representative histological images of transverse sections of diaphragm muscle stained with Picrosirius Red (a–d) or Haematoxylin and Eosin (e–h). (i–k) Summary data showing the relative area of collagen deposition (i), relative area of infiltration of inflammatory cells (j) and relative density of centrally nucleated cells (k). Values are expressed as box and whisker plots (median, 25th−75th percentile and scatter plot) and were compared statistically using one‐way ANOVA with Tukey's multiple comparisons *post hoc* test (Prism v.10.3.1). Values of *p* < 0.05 are reported. Abbreviations: PRED, α‐methylprednisolone; PREDNAC, α‐methylprednisolone plus *N*‐acetyl cysteine.

Representative histological images for diaphragm muscle from wild‐type, *mdx*, *mdx* + PRED and *mdx* + PREDNAC groups are presented in Figure 10. Collagen deposition in the *mdx* diaphragm was significantly increased (*p *< 0.0001), and neither drug treatment significantly reduced diaphragm collagen deposition. The relative area of immune cell infiltration (*p *< 0.0001) and the density of centrally nucleated myofibres were significantly increased in the *mdx* diaphragm (*p *< 0.0001). PRED treatment alone did not significantly reduce immune cell infiltration or central nucleation. However, PREDNAC treatment significantly reduced immune cell infiltration (*p *< 0.0001) and central nucleation (*p* = 0.0011) in the *mdx* diaphragm.

### 3.6 Diaphragm muscle contractile function ex vivo

**FIGURE 11:**
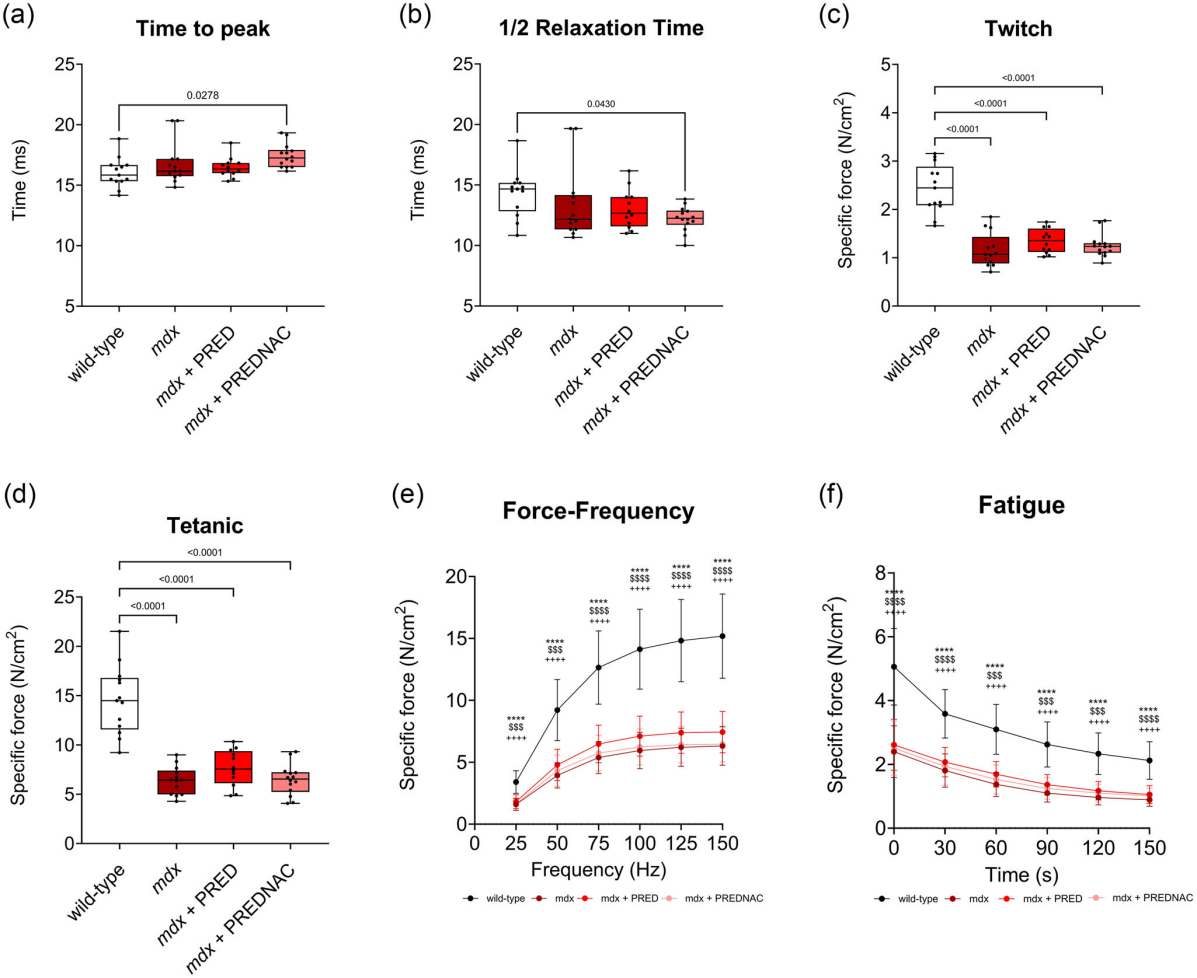
*Ex vivo* assessment of diaphragm time to peak (a), half‐relaxation time (b), twitch force (c), tetanic force (d), force–frequency relationship (e) and fatigue characteristics (f), in wild‐type (*n* = 13), *mdx* (*n* = 13), *mdx* + PRED (*n* = 12) and *mdx* + PREDNAC (*n* = 14) groups. Values are expressed as box and whisker plots (median, 25th−75th percentile and scatter plot) or mean ± SD (fatigue and force–frequency plots). Force and contractile kinetics data were compared statistically using a one‐way ANOVA with Tukey's multiple comparison *post hoc* test. The fatigue and force–frequency relationships were compared by two‐way ANOVA with Tukey's multiple comparisons *post hoc* test (Prism v.10.3.1). Values of *p* < 0.05 are reported for time to peak, half‐relaxation time, twitch force and tetanic force. Force–frequency and fatigue data are shown as the mean ± SD and were compared statistically using two‐way ANOVA with Tukey's *post hoc* test. * Denotes *mdx* different from corresponding wild‐type group: ^**^ *p* < 0.01, ^***^ *p* < 0.001 and ^****^ *p* < 0.0001. $ Denotes *mdx* + PRED different from corresponding *mdx* group: ^$^ *p* < 0.05, ^$$^ *p* < 0.01, ^$$$^ *p* < 0.001 and ^$$$$^ *p *< 0.0001. + Denotes *mdx* + PRED different from corresponding *mdx* group: ^+^ *p* < 0.05, ^++^ *p* < 0.01, ^+++^ *p* < 0.001 and ^++++^ *p *< 0.0001. Abbreviations: PRED, α‐methylprednisolone; PREDNAC, α‐methylprednisolone plus *N*‐acetyl cysteine.

Figure 11 shows summary data for diaphragm twitch time to peak, twitch half‐relaxation time, twitch force, tetanic force, the force–frequency relationship and fatigue characteristics for wild‐type, *mdx*, *mdx* + PRED and *mdx* + PREDNAC groups. Diaphragm force was significantly compromised in *mdx* mice, and this was not rescued by either of the two drug treatments.

## 4 DISCUSSION

The major findings of this study are as follows: (1) chronic PREDNAC treatment reduced immune cell infiltration and central nucleation in *mdx* diaphragm; (2) chronic PRED and PREDNAC increased peak *mdx* diaphragm EMG activity, and PRED alone increased parasternal intercostal EMG activity; (3) chronic PRED or PREDNAC did not restore force‐generating capacity of the *mdx* diaphragm; (4) chronic PRED or PREDNAC did not affect *mdx* inspiratory pressure‐generating capacity; and (5) chronic PRED or PREDNAC had no effect on ventilation and ventilatory responsiveness in *mdx* mice.

We gave PRED once weekly for 3 months to determine whether that would elicit beneficial effects on respiratory muscle quality and function without the deleterious effects associated with chronic daily administration. In separate animals, we administered weekly PRED combined with daily NAC (a dietary antioxidant, anti‐inflammatory and anti‐fibrotic agent) to investigate whether this would further ameliorate dystropathology in the *mdx* diaphragm over the period of 1–4 months of age.

Structural remodelling within the diaphragm is evident very early in the *mdx* mouse model. This remodelling includes increased collagen deposition within the diaphragm (indicative of fibrosis), an increased number of centrally nucleated cells (marker of fibre regeneration) and infiltration of the muscle by immune cells (response to injury). We have previously described that at 1 month of age, collagen deposition in the diaphragm is equivalent in *mdx* and wild‐type mice, but by 4 months of age there is a significant increase in collagen deposition in the *mdx* diaphragm and increased levels of the canonical profibrotic signalling factor transforming growth factor‐β1 (O'Halloran et al., 2023). Both PRED and NAC work by anti‐inflammatory actions. Daily PRED has been reported to be effective in reducing immune cell infiltration within skeletal muscle when administered over a short period of time, but with prolonged daily use PRED can become ineffective and can even cause muscle atrophy (Quattrocelli, Barefield et al., 2017).

In our study, weekly PRED alone had no effect on diaphragm fibrosis, immune cell infiltration and central nucleation in the *mdx* mouse model, indicating that the progression of the disease advances too rapidly for a single dose of PRED once weekly to be effective in ameliorating these effects. PREDNAC as a combination therapy likewise did not significantly reduce collagen deposition in the *mdx* diaphragm. However, PREDNAC significantly decreased the relative area of immune cell infiltration and the relative density of centrally nucleated myocytes in the diaphragm. These reductions show that combined weekly glucocorticoid and daily antioxidant are effective in partly reducing the inflammation and injury‐related regeneration in the diaphragm of the *mdx* mouse model of DMD. However, these improvements are not sufficient to offset muscle weakness and fibrosis.

Peak diaphragm muscle force is reduced in the *mdx* mouse model as early as 1 month of age (O'Halloran et al., 2023) and perhaps sooner. The present study confirms weakness in the diaphragm at 4 months of age. Diaphragm force was determined across a range of stimulus intensities, and our study demonstrates that neither PRED nor PREDNAC treatment rescues diaphragm muscle force in the *mdx* mouse model. This demonstrates that a weekly treatment regimen alone or in combination with daily antioxidant is not effective in ameliorating the loss of force‐generating capacity in the *mdx* mouse model of DMD. This outcome was evident despite reduced inflammation within the *mdx* diaphragm with PREDNAC treatment, showing that the magnitude of the anti‐inflammatory effect was not functionally relevant.

Peak EMG activity in the *mdx* mouse model is significantly reduced at 1 month of age, and this reduced EMG activity persists to 16 months of age (O'Halloran et al., 2023). A reduction in respiratory EMG activity is evident as a reduction in the amplitude of motor unit action potentials owing to attrition of large motor units and/or dysfunction in the neuromuscular junction. The present study revealed a reduction in *mdx* EMG activity compared with wild type within three of the eight respiratory muscles that were investigated. EMG activity was recovered in the parasternal intercostal muscle with PRED alone but not with PREDNAC. Diaphragm EMG activity was increased with both PRED and PREDNAC treatments compared with *mdx*, with values increasing more than wild type. The partial recovery of diaphragm and parasternal EMG activities is interesting. These two muscles carry a considerable burden of breathing and are likely to be the muscles most injured by contraction‐induced injury in *mdx* mice. The anti‐inflammatory effect of treatment in the diaphragm (and presumably parasternal) muscle might have improved neuromuscular integrity, owing to improvements in muscle quality, but this is speculative. Neither PRED nor PREDNAC influenced any of the accessory muscles of breathing, where all values were equivalent to *mdx* mice.

At 1 month of age, peak inspiratory pressure‐generating capacity is reduced in *mdx* mice compared with wild‐type mice (O'Halloran et al., 2023). Paradoxically, at 4 months of age, pressure generation is greater in *mdx* mice compared with wild‐type counterparts (O'Halloran et al., 2023). The present study confirmed that *mdx* mice have a greater inspiratory pressure‐generating capacity than wild‐type mice. This adaptive response relates to diaphragm remodelling and compensation afforded by accessory muscles of breathing in early dystrophic disease (O'Halloran et al., 2023). Loss of this compensatory mechanism ensues in advanced dystrophic disease, underpinning the emergence of integrated respiratory system dysfunction (O'Halloran et al., 2023). Neither PRED alone nor combined PREDNAC treatment affected inspiratory pressure‐generating capacity (from baseline to peak activities) in the *mdx* mouse model of DMD despite the increase in EMG activity in the diaphragm.

The present study investigated baseline ventilation and ventilatory responsiveness to HcHx in conscious mice, and ventilation during baseline conditions, following bilateral vagotomy and subsequent exposure to HcHx in anaesthetized mice. Our results confirm previous work (O'Halloran et al., 2023) indicating that despite diaphragm injury and weakness, *mdx* mice maintain normal breathing and retain fully the ability to increase breathing in response to challenges across the ventilatory range in early disease. Weekly PRED or combined PREDNAC treatment had no effect on breathing and metabolism in *mdx* mice.

In summary, the PRED dosing strategy (once weekly 0.8 mg/kg i.p.) used in this study was ineffective in offsetting major facets of the disease, with no changes to diaphragm muscle structure or function. Co‐administration of PREDNAC (once weekly PRED 0.8 mg/kg i.p. and 1% daily NAC in drinking water) caused a reduction in inflammation within the diaphragm muscle. However, diaphragm function was still impaired, indicating that the anti‐inflammatory effect was not functionally relevant and was ultimately inadequate. PREDNAC also provoked an increase in diaphragm EMG activity; however, pressure‐generating capacity was unaffected. It is evident that disease progression was too great to overcome by means of the current treatment strategy.

Our approach provided a comprehensive screening of respiratory performance in the *mdx* model, which is a strength. Breathing and chemoresponsiveness, in addition to integrated respiratory performance and diaphragm muscle force were assessed in all animals. Nevertheless, there are limitations of the study. It would have been useful to have concurrent information on limb muscle function to establish whether the lack of effect on the diaphragm reported in the study was specific to the respiratory muscle. We did not confirm the previous findings of Quattrocelli, Barefield et al. (2017). We studied one dose of PRED and NAC and chose a 3 month intervention for study, representing a dynamic period in the elaboration of a disease phenotype, but we acknowledge that different doses over different periods would be an added strength to the study. In our study, we did not investigate cross‐sectional area or the thickness of the diaphragm because there was no increase in force in treated *mdx* diaphragms. We reason that there most probably was no difference in these parameters given the relationship between form and function. This differs from the findings of Quattrocelli, Barefield et al. (2017), who reported increased diaphragm cross‐sectional area and thickness after weekly glucocorticoid treatment (but atrophy when used daily), but the two studies differ in several ways. We used the traditional *mdx* mouse, started treatment at 1 month of age for a period of 3 months using α‐methylprednisolone at 0.8 mg/kg, and studied diaphragm but not limb muscle function. Quattrocelli, Barefield et al. (2017) used the more fibrotic D2.*mdx* model, started treatment at 6 months of age for a period of 1 month using prednisone at 1.0 mg/kg, and studied limb but not diaphragm muscle function.

Our study revealed that although weekly PRED has previously been shown to have beneficial effects in limb muscle, it is ineffective in exerting any functionally beneficial effect on the respiratory muscles. Previous studies investigating glucocorticoids and NAC in models of DMD have explored numerous dosing strategies and dose durations, and they provide a broad list of outcome measures that are not focused on the respiratory system but instead mostly on limb muscle form and function. Quattrocelli, Barefield et al. (2017) showed that prednisone or deflazacort treatment (1 mg/kg i.p.) once weekly for 4weeks in 6‐month‐old D2.*mdx* mice was effective in diminishing some of the harmful side‐effects of the normal daily dosing regimen, showing improved grip strength, greater endurance to exercise and increased limb tetanic force. Hartel et al. (2001) showed that prednisone treatment (1 mg/kg, i.p.) daily for 8 weeks, starting at 4 weeks of age, reduced the levels of transforming growth factor‐β1 and hydroxyproline concentrations, and increased collagen crosslinking in *mdx* diaphragm muscle. Keeling et al. (2007) showed that prednisolone (5 mg/kg, orally) twice weekly (consecutive days), for 2 years, resulted in improved survival and forelimb force‐generating capacity. Concerning the potential benefits of NAC, Burns et al. (2019) showed that 1% NAC in the drinking water for 2 weeks rescued diaphragm function by increasing force‐generating capacity and by reducing fibrosis and immune cell infiltration. Pinniger et al. (2017) showed that 2% NAC for 6 weeks improved *mdx* grip strength and force in the extensor digitorum longus muscle, with evidence of a reduction in muscle inflammation and oxidative stress. Terrill et al. (2012) found that 1% NAC treatment for 1 week prevented exercise‐induced myofibre necrosis, with a reduction in creatine kinase activity, in *mdx* mice. It is tempting to consider that a more aggressive dosing strategy might be effective (and required) for respiratory muscles, but the findings by Quattrocelli, Barefield et al. (2017) that daily dosing for a period of 1 month results in atrophic signalling in D2.*mdx* diaphragm suggest that this would be associated with diaphragm dysfunction. This suggests that studies exploring dosing regimens less frequent than daily but more frequent than weekly are worthy of pursuit.

## 5 CONCLUSION

The primary cause of death in patients with DMD is cardiorespiratory failure. This draws sharp focus to the need for optimal therapies to support respiratory system performance across the natural history of the disease. Our study reveals that weekly dosing with glucocorticoids might be suboptimal in support of breathing, suggesting that more frequent dosing might be required, perhaps owing to the higher duty cycle of respiratory muscles, which are rhythmically active throughout the day, in comparison to the limb muscles, which poses challenges for the determination of optimal glucocorticoid dosing in the clinic. Further studies examining the efficacy of different dosing strategies in preclinical models are warranted.

## AUTHOR CONTRIBUTIONS

Michael N. Maxwell: administration of PRED and PREDNAC; acquisition of whole‐body plethysmography data; acquisition of diaphragm histology data; data and statistical analysis and interpretation of data; preparation of figures and drafting of the manuscript. Ben T. Murphy: acquisition of force data; data and statistical analysis; preparation of figures; drafting of the manuscript. Fiona B. McDonald: supervision, design, editing andcritical review of the manuscript. Ken D. O'Halloran: acquisition of funding; experimental design; supervision; acquisition of EMG and pressure data; editing and critical review of the manuscript. All authors have approved the final version of the manuscript and agree to be accountable for all aspects of the work in ensuring that questions related to the accuracy or integrity of any part of the work are appropriately investigated and resolved. All persons designated as authors qualify for authorship, and all those who qualify for authorship are listed.

## CONFLICT OF INTEREST

None declared.

## Data Availability

Most data are provided in the manuscript as individual data points. All data are available upon reasonable request.
